# Effects of 5-Year Exercise Training on Cognition in Older Adults: 10-Years Follow-Up from the Generation 100 Study

**DOI:** 10.1186/s40798-025-00956-0

**Published:** 2025-12-07

**Authors:** Jenny Bakken, Daniel E. Brissach, Emma M. L. Ingeström, Sindre Midttun, Tara L. Walker, Dorthe Stensvold, Atefe R. Tari

**Affiliations:** 1 The Cardiac Exercise Research Group at the Faculty of Medicine and Health Sciences, Department of Circulation and Medical Imaging, Norwegian University of Science and Technology, Trondheim, Norway; 2 Department of Neurology and Clinical Neurophysiology, St Olavs University Hospital, Trondheim, Norway; 3 Department of Cardiology, St Olavs University Hospital, Trondheim, Norway; 4 Clem Jones Centre for Ageing Dementia Research, Queensland Brain Institute, The University of Queensland, Brisbane, Australia; 5 Department of Thoracic Medicine, Clinic of Thoracic and Occupational Medicine, St Olavs University Hospital, Trondheim, Norway

**Keywords:** Cardiorespiratory fitness, Cognition, Generation 100, Objectively measured VO_2peak_, Older men and women

## Abstract

**Background and aim:**

The rapid aging of the global population is expected to lead to an increase in the incidence and prevalence of neurodegenerative diseases. Endurance exercise training is considered one of the most effective forms of prevention against neurodegenerative diseases. This study investigated the effects of a 5-year exercise training intervention at varying intensities on cognitive function in healthy older adults.

**Methods:**

1486 healthy older men (*n* = 735) and women (*n* = 751) aged 70–77 years from Trondheim, Norway, were randomly assigned to high-intensity interval training (HIIT, *n* = 382), moderate-intensity continuous training (MICT, *n* = 367), or a control group (*n* = 737) following the national physical activity (PA) guidelines. Cognitive function was assessed using the Montreal Cognitive Assessment (MoCA) score after 3, 5 and 10 years. A linear mixed model was used to estimate the effect of the exercise intervention between the groups. Measures of physical health, such as cardiorespiratory fitness (CRF), and PA level was also measured.

**Results:**

After 10 years, we observed no significant differences in MoCA scores between the exercise training groups and the control group, despite variations in exercise intensity and good adherence to the exercise interventions. The HIIT group consistently showed higher CRF throughout the study period.

**Conclusions:**

While structured aerobic exercise training over 5 years improved physiological health markers in healthy older adults, its impact on mitigating cognitive decline was limited. Further long-term training studies in different populations are needed to better understand the link between exercise training and cognition in older adults.

*Trial Registration* The Generation 100 Study is registered at ClinicalTrials.gov (Identifier: NCT01666340), registered on 21 August 2012: https://clinicaltrials.gov/study/NCT01666340

**Supplementary Information:**

The online version contains supplementary material available at 10.1186/s40798-025-00956-0.

## Key Points

- A 5-year structured endurance training intervention did not result in significantly improved cognitive outcomes in healthy older adults, as measured by MoCA scores over a 10-year period.
- Participants in the high-intensity interval training (HIIT) group maintained higher CRF levels throughout the study, confirming the physiological benefits of the training protocol.
- The cohort consisted of older adults with relatively high baseline fitness levels, which may have limited the observable cognitive effects of additional exercise, highlighting the need for future studies in populations with lower baseline fitness.

**Supplementary Information:**

The online version contains supplementary material available at 10.1186/s40798-025-00956-0.

## Introduction

 The preservation of cognitive function at old age is important for public health and individual well-being [1]. As the global population ages rapidly, the incidence of cognitive decline and mild cognitive impairment (MCI), and associated neurodegenerative conditions such as dementia, is increasing [1]. This trend poses significant challenges to affected individuals, their families, and healthcare systems worldwide [2]. Despite extensive research, the battle against cognitive decline remains arduous, with no definitive cure in sight [2]. Alzheimer’s disease is the most common form of dementia, accounting for about 70% of all dementia cases [1]. Recent data has shown that deaths from Alzheimer’s has increased dramatically (140.9%) from the year 2000 to 2021, whereas deaths from for example heart disease decreased by 2.1% in the same period [1]. Retrospective studies have estimated that about one-third MCI cases will eventually progress into dementia [3, 4]. This underscores the urgency of exploring preventive strategies to mitigate the risk or delay the onset of such conditions. Adopting a healthy lifestyle is proposed to be helpful and has been explored as a preventative or therapeutic strategy. Among the various lifestyle interventions examined, physical activity (PA) and exercise training have emerged as promising avenues due to their accessibility, low cost, and potential for wide-reaching impact [5–7].

While age and genetics are major and non-modifiable risk factors for dementia, the latest Lancet Commission reported that approximately 45% of dementia cases can be prevented by addressing 14 modifiable risk factors [5]. Observational studies indicate that PA could delay the decline in cognitive function in individuals at risk of, or diagnosed with, dementia [8–10], with aerobic exercise training that increases cardiorespiratory fitness (CRF) potentially exerting the most favorable effect [11]. Of interest, Blondell et al. found in their systematic review and meta-analysis of 47 longitudinal cohorts of adults aged 40 years and older, generally cognitively healthy at baseline, that higher levels of PA were associated with a reduced risk of cognitive decline with increasing age [8]. In a meta-analysis, Northey and colleagues, explored whether exercise training effectively improves cognitive functions in adults over 50 years of age [12]. They reported that physical exercise training programs enhance cognitive performance in older adults, irrespective of their initial cognitive condition. Importantly, few long-term randomized controlled trials (RCTs) examining the effect of exercise on cognition in older adults have been conducted.

A sub-study of the Generation 100 Study by Zotcheva et al. found no effect of 5-years of exercise training on cognitive function measured using the Montreal Cognitive Assessment scale (MoCA). Further they investigated the association between 5 years of exercise training and changes in CRF with changes on cognitive performance among 945 of the Generation 100 participants [13]. They found no effect of 5-years of exercise training on cognitive function measured as MoCA score. Maintaining or improving CRF from baseline to the 5-year follow-up, where an increase in CRF was defined as ≥ 3.5 mL/kg/min, was associated with higher MoCA scores compared to those who decreased in CRF over the same period. Other RCTs show inconsistent results, with some finding positive effects of exercise training on cognition among older adults [14], and others reporting no evidence of such an effect [15]. Therefore, further research, particularly with longer study durations is required.

By utilizing data from the Generation 100 Study, the aim of this study was to determine the effects of 5 years of systematic exercise training at two different exercise intensities, moderate-intensity continuous exercise (MICT) and high intensity interval training (HIIT) on cognitive function measured as MoCA scores (at year 3, year 5 and year 10 follow-up testing) in comparison to a control group following the national guidelines for PA. We hypothesized that while all study groups would experience a cognitive decline, those in the exercise training groups would exhibit a slower decline, suggesting that age-related cognitive decline might be counteracted by exercise training. Given that previous studies have indicated an increased risk of cognitive decline in individuals with a history of cardiovascular disease (CVD) [16, 17], we also examined whether participants with established CVD at baseline exhibited differences in cognition compared to those without pre-existing CVD.

## Methods

### Study Population

**Fig. 1:**
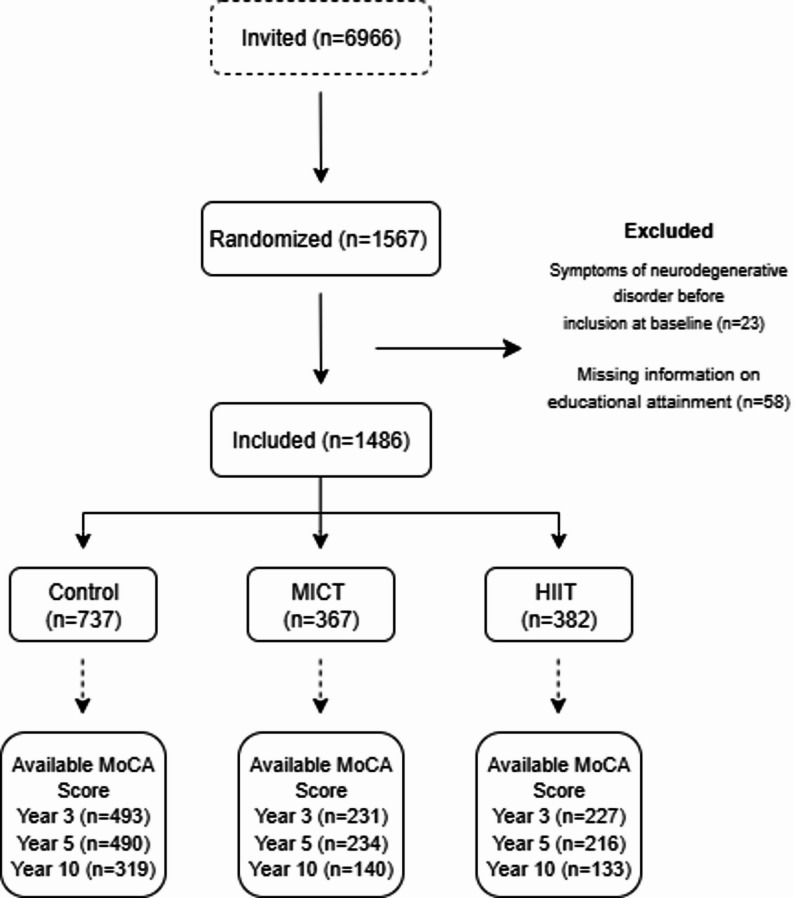
Flowchart study cohort. *MICT* moderate-intensity continuous training, *HIIT* high intensity interval training

This study draws upon data from the Generation 100 Study, a randomized controlled trial and the largest long-running exercise intervention among older adults to date [18]. The primary aim of the Generation 100 Study was to explore whether 5 years of supervised exercise training—HIIT or MICT—could reduce mortality and morbidity compared with a control group following national PA recommendations. Participants were recruited from Trondheim, Norway, between August 2012 and June 2013, with eligibility criteria including individuals born between 1936 and 1942 (*n* = 6966), and being able to perform an exercise intervention. In total, 1567 older adults (790 women) aged 70–77 years participated. The recruitment procedures have been described in detail previously [19], and the inclusion and exclusion criteria are summarized in Box 1. In this sub-study, we excluded participants who had missing information on educational level (*n* = 58), and participants with documented or clinically verified neurodegenerative disorders before inclusion at baseline (e.g., dementia, Parkinson’s disease, or other diagnosed neurodegenerative conditions) (*n* = 23). In total, 1486 participants were included in the final analysis on the effect of exercise training on cognition (Fig. 1).

**Box 1:** List of inclusion and exclusion criteria for the Generation 100 Study

**Inclusion criteria:** Born during 1936–1942, with a permanent address in Trondheim, Norway. Able to complete the exercise program (determined by the researchers). **Exclusion criteria:** Illness or disabilities that preclude exercise or hinder completion of the study. Uncontrolled hypertension. Symptomatic valvular, hypertrophic cardiomyopathy, unstable angina, primary pulmonary hypertension, heart failure or severe arrhythmia. Diagnosed dementia. Cancer that makes participation impossible or exercise contraindicated. Considered individually, in consultation with physician. Chronic communicable infectious diseases. Test results indicating that study participation is unsafe. Participation in other studies conflicting with participation in Generation 100.	

### Design

Participants were invited to testing at baseline (before randomization) and after 1, 3, 5, and 10 years. This included assessment of CRF, clinical examinations and cognitive testing. Participants also filled out a questionnaire regarding different aspects of health and lifestyle. For full details, see Stensvold et al. [19].

After baseline testing, participants were randomized in a 1:1 ratio to either an exercise training group or a control group. The exercise training group was further randomized into a MICT or HIIT group. The randomization was stratified by sex and cohabitation status.

### Exercise Training Intervention

The control group was instructed to follow the existing national guidelines for PA, which recommend 30 min of moderate to vigorous PA daily [20]. Participants in the MICT and HIIT group were prescribed two weekly exercise training sessions for 5 years. They could perform sessions individually or by attending organized group sessions held twice per week. In addition, every sixth week, both exercise groups participated in a mandatory supervised indoor cycling session with exercise physiologists to monitor and ensure proper exercise intensity. The MICT group engaged in 50-min continuous exercise training sessions twice a week, maintaining 70% of peak heart rate. The HIIT group completed two weekly 40-min sessions of HIIT, involving 4-min intervals at 85–95% of peak heart rate interspersed with 3-min active breaks [19]. Exercise intensity was individually based on each participant’s peak heart rate obtained from the cardiopulmonary exercise test. Adherence to the prescribed training sessions was assessed by validated questionnaires, as described in detail by Stensvold et al. [19]. None of the participants were offered supervised exercise training from year 5 testing until the year 10 follow-up testing.

### Cognitive Assessment

Cognitive assessment of the participants was done year 3, year 5 and year 10, and conducted using the Norwegian translation (version 8.1) of the MoCA, a tool designed for a rapid assessment of overall cognitive health [21]. It aims to identify MCI by examining abilities across several domains, including visuospatial/executive functions, naming, memory, attention, language, abstraction, delayed recall, and orientation. Higher scores, indicate better cognitive function, with a maximum possible score of 30 points [21]. The test was administered to all participants by personnel who had undergone uniform training and adhered to a consistent testing procedure without prior knowledge of the participants’ group randomization [19]. The MoCA test was not conducted at baseline or at year 1 follow-up testing due to limited availability of time and personnel resources.

We used the raw MoCA score, without adding an extra point for less than 12 years of education [21, 22]. As the MCI cut-off of 26 points has been shown to generate a high number of false positives [23, 24], we followed the approach recommended by Engedal et al. [25] to calculate a z-score, a statistical measure that quantifies how much an individual’s MoCA score deviates from the normative mean while accounting for age, sex, and educational background. First, we computed the normative MoCA scores for all participants. This involved determining an expected range of scores derived from a large, cognitively healthy, age-matched reference population, which served as the basis for interpreting individual MoCA scores. To estimate these normative MoCA scores, we applied a regression model that included coefficients for sex, education, age, and the interaction between age and the other variables [25]. The specific values used in the model are provided in Table S4A. Second, each participant’s observed MoCA score was compared to their predicted MoCA score from this regression model. The z-score was calculated using the following formula: $\:\frac{MoCA-\widehat{MoCA}}{2.085}$, where MoCA represents the observed score and $\:\widehat{MoCA}$ is the estimated MoCA score from the regression model. This z-score quantifies how many standard deviations (SD) an individual’s MoCA score deviates from the expected mean, accounting for age, sex, and educational background [25].

### Assessment of Cardiorespiratory Fitness

A cardiopulmonary exercise test was undertaken using ergospirometry with either Oxycon Pro (Erich Jaeger, Hoechberg, Germany), or Cortex Metamax II (Leipzig, Germany) to assess CRF. The same system was used consistently for each individual. The test was performed on a treadmill or a cycle ergometer, depending on participant preference and health status. The majority of participants performed the test on a treadmill, while about 3–4% used a cycle ergometer at each testing time point. Participants with a history of heart diseases were simultaneously monitored with a 12-lead electrocardiogram (Custo Cardio 100 BT, Custo Med GmbH, Ottobrunn, Germany) using the American Heart Association’s exercise testing guidelines for patients with heart diseases [26]. A full description of the test protocol is provided elsewhere [19]. In summary, after a warm-up phase, an individualized test protocol was applied, during which the workload was gradually increased approximately every 1.5 min or when VO_2_ stabilized. This procedure continued until voluntary exhaustion (VO_2peak_) or until maximal oxygen uptake (VO_2max_) was reached. Heart rate was continuously monitored using a RS100 (Polar Electro Oy, Kempele, Finland). Maximal heart rate recorded during the test was used to determine exercise training intensity. A test was considered maximal when the participant continued until exhaustion, and VO_2_ did not increase by more than 2 mL/kg/min between two 30-s epochs (i.e., a leveling-off of VO_2_ despite increased workload), combined with a respiratory exchange ratio (RER) of ≥ 1.05. Since approximately 40% of the participants did not achieve a true VO_2max_ at each testing time point (baseline, year 1, 3 and 5), we use the collective term VO_2peak_ as the measure of CRF throughout this paper.

### Cardiovascular- and Neurodegenerative-Disease Follow-Up

Participants in the study were assessed for cardiovascular-, and neurodegenerative disease before inclusion at baseline by reviewing their medical records. In this study, we excluded participants with symptoms of neurodegenerative disorder to maintain integrity of the data. Participants with established CVD before inclusion at baseline were included, coincide with separate analysis of these participants.

### Descriptive Data

Participants completed a questionnaire regarding their health, socioeconomic status, and lifestyle. From this, we obtained information on age, sex, cohabitation status, educational attainment, daily activity level, smoking status, hypertension, diabetes mellitus, self-reported good memory and family history of dementia. Blood pressure was measured with a Philips IntelliVue MP50 (Philips medizin systeme, Boeblingen, Germany). Hypertension was defined as blood pressure ≥ 140/90 or self-reported use of blood pressure medication. Venous blood samples for biochemical analyses were obtained from an antecubital vein (≥ 8 h postprandial) High cholesterol was defined as serum total cholesterol > 7.8 mmol/L. High serum triglycerides was defined as triglycerides > 1.7 mmol/L. Low serum high density lipoprotein (HDL) was cholesterol defined as < 1.0 mmol/L (men) and < 1.3mmol/L (women). Devices and laboratories used are under the quality control at St. Olavs University Hospital, Trondheim, Norway where quality controls are frequency performed. Age and sex were verified through national databases.

### Statistical Analysis

Descriptive statistics are presented as mean and standard deviation (SD) for continuous variables or as numbers and percentages (%) for categorical variables. The data were reviewed for discrepancies and examined for a normal distribution pattern by visual review of Q–Q plots. All the variables under consideration, apart from the questionnaire data, followed a normal distribution. Between group differences at the different timepoints were assessed using a student’s t-test, and one-way ANOVA for 3 groups. Changes within groups (i.e. change from baseline to 1-year testing in the intervention groups) were assessed using a paired sample t-test. Results are reported as mean, SD and p-values and with 95% confidence intervals (CI) where relevant. All statistical tests were two-sided, and significance level was set at 5%.

To evaluate the between-group effects of the exercise training intervention on MoCA scores and CRF, we employed a Linear Mixed Model (LMM). The mixed model accounts for missing data by including participants with incomplete data from one or more follow-up time points. These participants contribute to the estimates at available time points, albeit with reduced weight compared to those with complete data. Consequently, no imputation of missing data was necessary when using this method.

The model was adjusted for sex and cohabitation status (variables used in the stratified randomization) as well as age at inclusion. Baseline values of the outcome variables were included in the model as covariates, following the methodology and rationale described by Twisk et al. [27]. This approach allows for direct interpretation of the between-group effects as the estimate for the group × time interaction term. MoCA assessments from year 3 testing was thus used as baseline for assessing the trajectory of cognition over three different timepoints: from year 3 to year 5 follow-up, and year 3 to year 10 follow-up. Baseline CRF measurements were used to assess trajectories of CRF over the 5-year exercise training period, with assessments after year 1, year 3 and year 5.

For analyses of total MoCA scores, z-scores, and CRF, we applied two separate models: one comparing the HIIT and MICT groups individually with the control group, and another comparing the combined exercise training group (ExComb, comprising both HIIT and MICT participants) with the control group. Notably, the z-score analysis was performed unadjusted, as the z-scores were already standardized for gender, education, and age during calculation. Participants were included as random intercepts in the model, with all outcome variables separately considered as dependent variables. All analyses in this study were performed using IBM SPSS Statistics, Version 29.0.1.0 (SPSS Inc, Chicago USA).

## Results

### Baseline Characteristics

Baseline characteristics of the participants are presented in Table 1. A total of 1 486 participants were included in the study, 50.5% women. Mean (SD) age was 72.4 (2.04) years, BMI 26.0 (3.6) and VO_2peak_ 28.8 (6.5) mL/kg/min. Overall, men had higher VO_2peak_ at baseline than women, 31.4 (6.7) and 26.2 (5.0), respectively (*p* < 0.001).

**Table 1:** Characteristics at baseline classified by intervention group

	Control (*n* = 737)	MICT (*n* = 367)	HIIT (*n* = 382)
Mean (SD) age, years	72.4 (2.1)	72.3 (2.0)	72.5 (2.1)
Mean BMI (SD), kg/m^2^	25.9 (3.4)	25.9 (3.9)	26.2 (3.7)
Women	378 (51.3)	191 (52.0)	182 (47.6)
Cohabitation	542 (73.8)	262 (71.4)	294 (77.2)
Higher education (college/university)	369 (49.8)	192 (52.3)	195 (51.0)
Minimum 30 min PA daily	512 (73.1)	263 (75.8)	278 (75.3)
Mean (SD) VO_2peak,_ mL/kg/min	28.7 (6.4)	28.6 (6.6)	29.1 (6.4)
Daily smoker	40 (5.5)	28 (7.8)	14 (3.7)
History of CVD	125 (17.0)	71 (19.3)	56 (14.4)
Hypertension	462 (63.1)	220 (60.9)	210 (55.6)
High cholesterol	16 (2.2)	9 (2.5)	9 (2.4)
High serum triglycerides	89 (12.1)	50 (13.1)	45 (11.9)
Low HDL cholesterol	50 (6.8)	32 (8.8)	23 (6.1)
Mean LDL cholesterol (SD), mmol/L	3.42 (0.98)	3.35 (1.02)	3.38 (1.02)
Diabetes mellitus	36 (5.0)	19 (5.2)	23 (5.0)
Self-reported good memory	497 (67.4)	245 (66.8)	253 (66.2)
Family history of dementia	104 (16.1)	54 (16.4)	60 (17.3)

Data presented as n (%) unless stated otherwise

Hypertension is defined as blood pressure > 140/90 or self-reported use of blood pressure medication. High serum total cholesterol is defined as serum total cholesterol > 7.8 mmol/L. High serum triglycerides is defined as triglycerides > 1.7 mmol/L. Low serum HDL cholesterol defined as < 1.0 mmol/L (men) and < 1.3mmol/L (women)

*CVD* cardiovascular disease, *HDL* high-density lipoprotein, *HIIT* high intensity interval training, *LDL* low-density lipoprotein, *MICT* moderate-continuous training, *PA* physical activity, *VO*_*2peak*_ peak oxygen uptake

A total of 252 (151 in the control group; 71 in the MICT group; 56 in the HIIT group) had established CVD before inclusion at baseline. The baseline characteristics of these are presented in Table S2A.

### MoCA Scores at Year 3 Follow-Up Testing

There were no significant differences in total MoCA score among the groups at the first MoCA assessment at year 3 (*p* = 0.154). At year 3 follow-up testing, the mean MoCA score was 24.9 (SD: 3.2) in the total study population (Table S1A). The mean MoCA at year 3 for men and women was 24.8 (SD: 3.2) and 25.0, respectively (*p* = 0.222).

The mean MoCA score at year 3 for participants with established CVD at baseline was 24.5 (SD: 3.1) (Table S3A). This score did not significantly differ from the mean MoCA score of 25.0 (SD: 3.2) observed in participants without CVD at baseline (*p* = 0.100).

### Changes in MoCA Score

Overall, analysis showed that MoCA score decreased by 0.39 points (*p* = 0.002) from year 3 to year 5, and by 0.98 points (*p* < 0.001) from year 3 to year 10. The mean z-score tended to decrease between year 3 to year 5 (*p* = 0.07) but not from year 3 to year 10 (*p* = 0.31).

On average, women had 0.46 (95% CI 0.73– 0.84, *p* = 0.020) points higher MoCA scores than men over the study period. Higher age was associated with lower MoCA scores, with each additional year of age linked to a 0.13-point decline in MoCA score (95% CI − 0.22 to − 0.04, *p* = 0.006).

### Group Differences

Table 2 presents the results from the LMM examining the trajectory of MoCA scores at years 3, 5 and 10. No statistically significant differences in MoCA scores were observed between the exercise training groups and the control group at any time point. After 10 years, the MICT group had 0.41 points higher MoCA scores than the control group (95% CI − 0.08 to 0.90), the HIIT group 0.23 points higher (95% CI − 0.28 to 0.73), and the ExComb group 0.32 points higher (95% CI − 0.08 to 0.72), but none of these differences were statistically significant. For z-score, small, non-significant differences between the groups at year 3, year 5 and year 10 follow-up testing was observed (Table 2).

**Table 2:** Results from the LMM showing estimated treatment effect as year × group interaction in the total study population

	Control	HIIT vs. CON	MICT vs. CON	HIIT vs. MICT	ExComb vs. CON
Total MoCA score					
Year 3	24.7 (3.3)				
Year 5	24.4 (3.4)	0.05 (− 0.39–0.47)	− 0.05 (− 0.45–0.35)	0.10 (− 0.38–0.58)	0.00 (− 0.33–0.33)
Year 10	24.3 (3.4)	0.23 (− 0.28–0.73)	0.41 (− 0.08–0.90)	− 0.18 (− 0.77–0.41)	0.32 (− 0.08–0.72)
z-score					
Year 3	− 0.51 (1.51)				
Year 5	− 0.57 (1.56)	− 0.02 (− 0.24–0.20)	− 0.14 (− 0.35–0.08)	0.05 (− 0.18–0.27)	− 0.01 (− 0.16–0.15)
Year 10	− 0.33 (1.57)	0.06 (− 0.20–0.32)	0.08 (− 0.17–0.33)	− 0.08 (− 0.36–0.20)	0.15 (− 0.42–0.34)
VO_2peak_					
Baseline	28.7 (6.4)				
Year 1	30.6 (6.9)	0.99 (0.48–1.50)^*^	0.21 (− 0.31–0.72)	0.78 (0.19–1.38) ^*^	0.60 (0.18–1.02)^*^
Year 3	29.3 (7.1)	1.12 (0.62–1.72)^*^	0.08 (− 0.47–0.63)	1.09 (0.47–1.73) ^*^	0.63 (0.18–1.08)^*^
Year 5	28.1 (6.7)	0.76 (0.19–1.34)^*^	0.06 (− 0.51–0.63)	0.71 (0.03–1.34) ^*^	0.41 (− 0.06–0.88)

Data are presented as an estimate with 95% CI

*CI* confidence interval, *SD* standard deviation, *LMM* Linear mixed model, *MICT* moderate-intensity continuous training, *HIIT* High-intensity interval training, *VO*_*2peak*_ peak oxygen uptake (mL/kg/min)

The observed decline in MoCA score among participants with established CVD at baseline was 0.04 points from year 3 to year 5, (*p* = 0.904) and 0.47 points from year 3 to year 10 (*p* = 0.264). Table 3 present the results from the LMM examining the trajectory of MoCA scores at year 3, year 5 and year 10 follow-up testing among those with established CVD at baseline. The was no observed effect of exercise training on cognition.

**Table 3:** Results from the LMM showing estimated treatment effect as year × group interaction among participants with established CVD at baseline

	Control	HIIT vs. CON	MICT vs. CON	HIIT vs. MICT	ExComb vs. CON
Total MoCA score					
Year 3	24.0 (3.2)				
Year 5	24.1 (3.1)	− 0.30 (− 1.11–0.52)	− 0.54 (− 1.58–0.50)	0.24 (− 0.72–1.20)	− 0.36 (− 1.13–0.41)
Year 10	23.7 (3.8)	− 0.18 (− 1.20–0.84)	0.69 (− 0.66–2.05)	− 0.87 (− 2.13–0.66)	0.03 (− 0.94–1.00)
z-score					
Year 3	− 0.84 (1.38)				
Year 5	− 0.81 (1.46)	0.26 (− 0.29–0.82)	− 0.18 (− 0.67–0.31)	0.44 (− 0.17–1.05)	− 0.01 (− 0.43–0.43)
Year 10	− 0.71 (1.74)	− 0.15 (− 0.94–0.63)	0.43 (− 0.22–1.07)	− 0.58 (− 1.43–0.28)	0.22 (− 0.35–0.76)

Data are presented as an estimate with 95% CI

*CI* confidence interval, *CVD* Cardiovascular disease, *SD* standard deviation, *LMM* Linear mixed model, *MICT* moderate-intensity continuous training, *HIIT* High-intensity interval training, *VO*_*2peak*_ peak oxygen uptake (mL/kg/min)

### Cardiorespiratory Fitness

For the total study population, the mean VO_2peak_ increased by 2.3 mL/kg/min (8.0%) from baseline to year 1 follow-up testing (*p* < 0.001). There was practically no change in mean VO_2peak_ over the 5-year exercise training intervention (28.8–28.5 mL/kg/min, 1.1% decline, not significant).

**Fig. 2:**
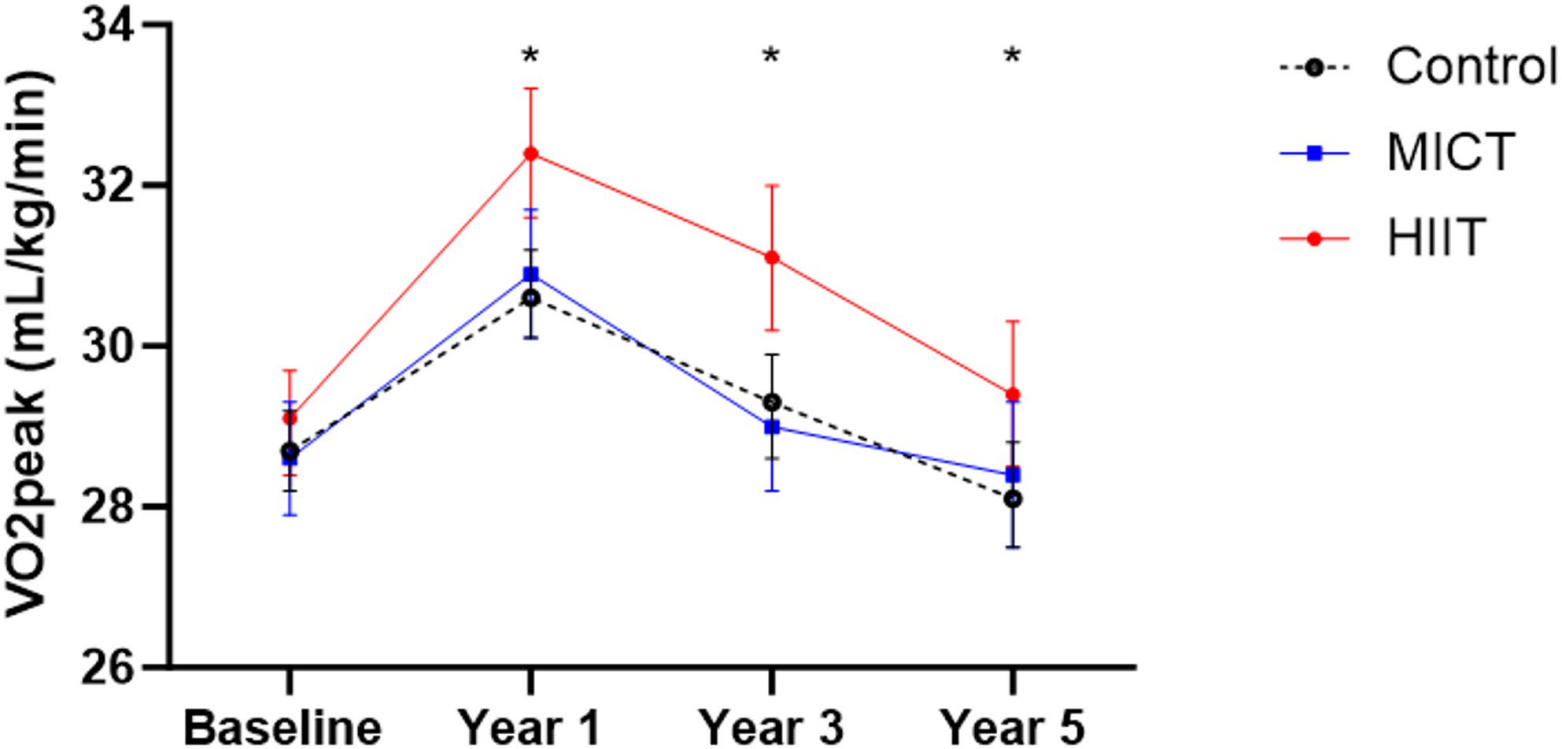
Changes in mean VO_2peak_ over 5 years in control, MICT, and HIIT. Data are presented as mean VO_2peak_ with 95% error bars in the different intervention groups from baseline, year 1, year 3 and year 5. *MICT* moderate-intensity continuous training, *HIIT* high intensity interval training. *= Significantly different from both control and MICT

The mean VO_2peak_ values for the intervention groups at different time points are presented in Table S1A. There were no significant differences in VO_2peak_ among the groups at baseline (*p* = 0.593). After 1 year of exercise training, VO_2peak_ increased in all groups (Fig. 2). The HIIT group had 1.0 mL/kg/min higher VO_2peak_ compared to the control group (*p* < 0.001) and 0.8 mL/kg/min higher CRF compared to the MICT group (*p* = 0.010) after 1 year of exercise training. No significant difference in VO_2peak_ after 1 year of exercise training in MICT vs. control was observed (*p* = 0.432) (Table 2). After year 3 and year 5, the HIIT group still showed a significantly higher VO_2peak_ compared to both the control group (*p* < 0.001 and *p* = 0.040, respectively) and the MICT group (*p* < 0.001 and *p* = 0.009, respectively). VO_2peak_ was significantly higher in the ExComb group compared to the control group at year 1 and year 3 (*p* = 0.005 and *p* = 0.006, respectively), but not at year 5 (*p* = 0.085) (Table 2).

### Adherence to Exercise Training Program and Physical Activity Patterns

After 1, 3, 5 and 10 years, respectively, adherence to the prescribed exercise training in our study sample was 71%, 67%, 68% and 59% in the MICT group, and 53%, 51%, 44% and 34% in the HIIT group. In the control group, the proportion following national guidelines for physical activity was 80%, 80%, 76% and 72% at 1, 3, 5 and 10 years, respectively. Among the controls, 25%, 28%, 24% and 23% reported to perform exercise training as HIIT at 1, 3, 5 and 10 years, respectively. Details on PA patterns and adherence are presented in Table S1B.

## Discussion

In the present study, we found that despite rigorous methodologies and significant differences in terms of CRF across the different exercise groups, no differences in cognition over the follow-up period of 10 years was observed.

### Cognitive Decline Over 7 Years

Overall, our analysis revealed a decline in MoCA score of 0.98 points over 7 years (from year 3 to year 10 follow-up testing). To date, there are limited data on longitudinal change in MoCA score in a generally healthy older aged population. However, our findings align with the suggested decline in Norwegian [25] and Swedish individuals [28], where studies have reported a comparable cognitive decline over a decade of ageing after the age of 65 years. In contrast, studies from other countries have reported a decline varying from 0.6 to 2.4 points in MoCA score over the same period [29–32]. These discrepancies may be attributed to population differences in important factors for MoCA such as socioeconomic and sociodemographic characteristics [25, 29]. Methodological variations, including differences in test administration and translation quality, could also contribute to the observed variation across countries [31, 33].

Our analysis indicated that women had a MoCA score 0.46 points higher than men. This is in line with other normative MoCA studies [29, 34]. Our observations also align with a study showing that sex differences in cognitive tests are stable over a period of up to 10 years in older adults [35]. Our observation that higher age was associated with lower MoCA scores also aligns with previous studies [34, 36].

### Effects of 5 Years of Exercise Training on Cognition

Although we found no significant effects of 5 years of exercise training on cognition, the ExComb group had a non-significant 0.32 points higher MoCA scores after 10-years, compared to control. Similarly, both the MICT and HIIT group had higher MoCA scores after 10-year, when analyzed separately (not statistically significant). Although the absence of baseline MoCA assessments limits the ability to capture early cognitive effects of the intervention, it is possible that subtle improvements occurred during the first years, particularly among participants in the HIIT group, who demonstrated higher CRF at both year 1 and year 3. MoCA scores showed a modest and comparable decline across all groups during the follow-up. However, the highly active control group, with high adherence to national physical activity guidelines and regular vigorous exercise, may have reduced between-group differences, potentially masking early or fitness-related cognitive effects.

Our results are consistent with other RCTs assessing the effects of exercise training on global- and domain-specific cognition. In 2021, a study by Zotcheva et al., also using data from the Generation 100 Study, that found no effect of exercise training on cognition [13]. That study included 945 of the participants and only looked at the year 5 MoCA results. Expanding on this, we had MoCA scores measured at three different time points and included all participants who were free from neurodegenerative disorders at baseline and had reported educational attainment, resulting in a total of 1486 participants in our study. Similar to the findings from both Zotcheva et al. [13] and the present study, the Lifestyle Interventions and Independence for Elders study found that a 24-month moderate-intensity PA intervention did not result in notable improvements in overall or specific areas of cognitive function when compared to a health education program among older, sedentary adults [37]. However, it is worth noting that the 1467 participants of the Lifestyle Interventions and Independence for Elders study were older, with a mean age of 78.9 years, and 141 of them had MCI at baseline. An umbrella review by Ciria et al. [38] evaluated the scientific evidence on the cognitive benefits of regular exercise training. They found small but positive effects, though the evidence was not strong enough to support definitive conclusions. This underscores the inconsistency of findings from RCTs and highlights the need for more rigorous research in this area. The inconsistent findings regarding exercise-related effects on cognition may partly reflect considerable heterogeneity across studies, including differences in training duration, exercise intensity, adherence, and participant characteristics such as baseline fitness or cognitive status. Additionally, cognitive outcomes vary widely across studies, with some assessing global cognition (e.g., MoCA) and others focusing on domain-specific tests, further complicating comparisons. Collectively, these methodological and population-related differences may contribute to the mixed evidence observed in the literature. Future studies may require longer follow-up periods to capture the sustained effects of exercise training on cognition. The MoCA is a brief, validated global screening tool well suited for large-scale population studies, but it may be less sensitive to subtle or domain-specific cognitive changes potentially induced by exercise. Extending the follow-up period alone may therefore not be sufficient to capture meaningful differences in cognitive outcomes. Future studies should include more comprehensive cognitive test batteries that assess specific domains such as executive function, processing speed, and memory, alongside objective measures of fitness and physical activity. Additionally, a clearer distinction between exercise training intensities is necessary to better understand their differential impact on cognitive outcomes. Furthermore, greater population representativeness is crucial, as many trials primarily recruit highly motivated individuals with preexisting training habits and thus limiting generalizability to the general population.

We believe several factors may have affected the possibility of detecting significant between-group differences. First, the participants that were included in the Generation 100 Study had better self-reported health, higher educational attainment and were more physically active compared to non-participants, as previously reported [19]. This selection bias may have influenced our results, as the healthy volunteer bias could have masked the effects of exercise training on cognition.

The mean MoCA score of 24.8 points at the first assessment in our study population is slightly lower than previously reported in Norway [25]. Engedal et al. analyzed data from 11,675 participants in the Trøndelag Health Study (HUNT) with a mean age of 76.2 years and found a mean MoCA score of 25.5 points [25]. Their study also showed that normative MoCA scores ranged from 22 to 27, depending on age, sex, and education level. To account for these factors, we calculated z-scores to assess how our participants’ cognitive performance deviated from the expected mean for their demographic characteristics based on the work by Engedal et al. [25]. Consistent with the lower total MoCA score observed in our study, the calculated z-score was also lower than the normative reference, with a mean of − 0.45 at the first assessment. This indicates that, on average, our study population scored 0.45 standard deviations below the expected for a population of the same age, gender and educational level. It is to be noted that a z-score of 0.45 below the expected is within normal ranges, as a cut-off of − 1.5 SDs is normally used as an indication of MCI [25].

Another possible explanation for why we did not see a difference between the groups may be a highly physically active control group. The control group reported to an overall high activity level and performed more HIIT than the MICT group. Combined with an adherence in the HIIT group of 53%, 51%, 44% and 34% after 1, 3, 5 and 10 years respectively, this likely influenced the lack of group differences in cognition in our study. Further, it is possible that the potential for cognitive benefits from exercise training may be diminished in aged individuals due to reduced brain plasticity [39].

### Effect of 5 Years of Exercise Training on CRF

CRF increased in all groups from baseline to year 1, with the HIIT group showing greater improvements following 1 year of exercise training compared to both the MICT and control group. From year 1 to year 5, all groups exhibited a linear decline of approximately 2% per year, which is consistent with the expected 20% decline over 10-years in older adults [40, 41]. Notably, due to the significant increase following the first year of exercise training, the mean CRF was 0.3 mL/kg/min higher after 5 years of exercise training, than at baseline in the HIIT group. Hence, the HIIT group carried the advantage of a higher improvement in CRF following 1 year of exercise training and maintained a higher CRF over 5 years.

Compared to one of the largest and most relevant reference data on CRF levels for our study population, our mean VO_2peak_ values are consistent with what previously has been reported in healthy Norwegian older adults aged >70 years [42]. However, compared to reference data from the US, the mean VO_2peak_ among both men and women in our study is significantly higher [43]. In fact, participants have the same VO_2peak_ levels as 50–60-year-old men, and 40–50-year-old women when compared to their reference values, respectively [43].

### Cardiovascular Disease

Participants with established CVD before inclusion at baseline did not show a significant difference in MoCA scores at the first assessment compared to those without CVD. Compared to normative data from 2653 participants in the Dallas Heart Study (mean score of 23.4) [30], the mean MoCA score was higher among individuals with CVD in our population (mean score of 24.5). The Dallas Heart Study included an ethnically diverse cohort assessed for factors contributing to the progression from normal health to cardiovascular risk. Notably, the mean age in their study was remarkably lower (50.3 years, compared to 72.4 years in our study).

### Strengths and Limitations

A key strength of the present study is its longitudinal and randomized design, allowing for assessment of the participants’ cognitive and exercise training trajectories over time. Another strength of the study is that exercise intensity was individually prescribed based on each participant’s peak heart rate from the cardiopulmonary exercise test, ensuring that both MICT and HIIT were accurately tailored to individual fitness levels and physiological capacity. An additional strength is the well-balanced distribution between men and women. Further, all testing was conducted using the same protocol each year, making our results comparable year-to-year.

The study also presents several limitations that could impact the interpretation of its results. The absence of MoCA testing at baseline and at year one restricts the ability to assess changes in cognition directly attributable to the exercise training interventions from the outset. Another limitation is the use of MoCA as the sole measurement of cognitive function. The highly active control group may also have made it difficult to detect between group differences. Additionally, adherence in the HIIT group, where only about half of the participants fully adhered to the exercise training protocol, may have further contributed to this. Although partly mitigated using an LMM, which allows for the inclusion of all available data, the loss to follow-up introduced a risk of attrition bias. This means that, despite conducting analyses by intention-to-treat, differences in participation rates at follow-up may introduce bias. Lastly, although the Generation 100 Study is one of the most comprehensive exercise training studies among older adults, the participants may represent a healthier proportion of the general older-aged population in Norway.

## Conclusions

This sub-study of the Generation 100 Study contributes to the complex narrative surrounding exercise training and cognition in older adults. While no significant differences were observed among different exercise training intensities over a 7-year period, the role of PA in mitigating cognitive decline remains a vital area of study. Continued research is essential to discern the nuances of how different types and intensities of exercise training might benefit or fail to influence cognitive health as part of aging. Ultimately, this line of inquiry holds the potential to inform public health strategies and individual practices aimed at preserving cognitive function in the aging population.

## Supplementary Information

Below is the link to the electronic supplementary material.

Supplementary Material 1.

## Data Availability

We are not permitted to share individual-level data from the current trial due to ethical and legal restrictions. However, we welcome collaborative research, and researchers may be granted access to analyzed data upon reasonable request. Data analyses must be conducted within our university in accordance with institutional and national data protection regulations.

## Abbreviations

BMI: Body mass index
CRF: Cardiorespiratory fitness
CVD: Cardiovascular disease
HIIT: High intensity interval training
LMM: Linear mixed models
MCI: Mild cognitive impairment
MICT: Moderate-intensity continuous training
MoCA: Montreal cognitive assessment test
PA: Physical activity
RCT: Randomized controlled trial
VO_2peak_: Peak oxygen uptake
