# Supplementary material for: Effects of 5-Year Exercise Training on Cognition in Older Adults: 10-Years Follow-Up from the Generation 100 Study
Source: Sports Med Open. 2025 Dec 7;11:153. doi: 10.1186/s40798-025-00956-0 (PMC12682729; doi:10.1186/s40798-025-00956-0)
Supplement: Supplementary file 1 — Supplementary Material 1. [file 40798_2025_956_MOESM1_ESM.docx]

# Appendix A

**Table S1A:** Mean MoCA, z-score group (based on available MoCA scores) and VO_2peak_ by intervention group

| Year | Control | MICT | HIIT | ExComb | Total |
| --- | --- | --- | --- | --- | --- |
| **Total MoCA score** |  |  |  |  |  |
| Year 3 | 24.7 (3.3) | 25.2 (3.1) | 25.0 (3.2) | 25.1 (3.1) | 24.9 (3.2) |
| Year 5 | 24.4 (3.4) | 24.6 (3.4) | 24.7 (3.1) | 24.6 (3.3) | 24.5 (3.3) |
| Year 10 | 24.3 (3.4) | 24.9 (3.1) | 24.6 (3.4) | 24.8 (3.3) | 24.5 (3.3) |
| **z-score** |  |  |  |  |  |
| Year 3 | -0.51 (1.51) | -0.26 (1.43) | -0.41 (1.42) | -0.34 (1.43) | -0.42 (1.5) |
| Year 5 | -0.57 (1.56) | -0.49 (1.56) | -0.46 (1.41) | -0.48 (1.49) | -0.53 (1.5) |
| Year 10 | -0.33 (1.57) | -0.06 (1.43) | -0.18 (1.58) | -0.12 (1.51) | -0.23 (1.5) |
| **VO_2peak_** |  |  |  |  |  |
| Baseline | 28.7 (6.4) | 28.6 (6.6) | 29.1 (6.4) | 28.8 (6.5) | 28.8 (6.5) |
| Year 1 | 30.6 (6.9) | 30.9 (6.7) | 32.4 (6.7) | 31.7 (6.7) | 31.1 (6.8) |
| Year 3 | 29.3 (7.1) | 29.0 (6.6) | 31.1 (6.9) | 30.1 (6.8) | 29.7 (7.0) |
| Year 5 | 28.1 (6.7) | 28.4 (6.7) | 29.4 (6.4) | 28.9 (6.4) | 28.5 (6.6) |

*Data presented as mean and SD. Total MoCA score is without 1 extra point a recommended by some versions of the test. SD, standard deviation; MICT, moderate-intensity continuous training; HIIT, High-intensity interval training; VO_2peak,_ peak oxygen uptake (mL/kg/min)*

**Table S2A:** Characteristics at baseline classified by intervention group among those with established CVD before inclusion at baseline

|  | Control (n=125) | MICT (n=71) | HIIT (n=56) |
| --- | --- | --- | --- |
| Mean (SD) age, years | 72.5 (2.2) | 72.3 (2.0) | 72.3 (2.1) |
| Mean BMI (SD), kg/m^2^ | 26.6 (3.3) | 25.6 (3.0) | 27.5 (4.2) |
| Women | 40 (32.0) | 18 (25.4) | 16 (28.6) |
| Cohabitation | 98 (78.4) | 55 (77.5) | 49 (87.5) |
| Higher education (college/university) | 63 (50.4) | 39 (54.9) | 36 (64.3) |
| Minimum 30 min PA daily | 74 (63.8) | 47 (69.1) | 44 (80.0) |
| Mean (SD) VO_2peak_, mL/kg/min | 27.6 (6.7) | 28.0 (7.2) | 28.8 (6.4) |
| Daily smoker | 7 (5.6) | 5 (7.0) | 1 (1.8) |
| Hypertension | 89 (71.8) | 45 (64.3) | 34 (60.7) |
| High cholesterol | 2 (1.6) | 0 (0) | 0 (0) |
| High serum triglycerides | 16 (12.9) | 10 (14.5) | 7 (12.5) |
| Low HDL cholesterol | 11 (8.9) | 8 (11.6) | 6 (10.7) |
| Mean LDL cholesterol (SD), mmol/L | 2.82 (1.02) | 2.73 (1.05) | 2.67 (1.05) |
| Diabetes mellitus | 11 (9.1) | 6 (8.6) | 6 (10.7) |
| Self-reported good memory | 76 (69.7) | 43 (69.4) | 35 (70.0) |
| Family history of dementia | 18 (15.9) | 8 (12.9) | 9 (18.4) |

*Data presented as n (%) unless stated otherwise. CVD cardiovascular disease; HDL, high-density lipoprotein; HIIT high intensity interval training; LDL, low-density lipoprotein; MICT moderate continuous training; PA physical activity; VO2peak peak oxygen uptake. Hypertension is defined as blood pressure >140/90 or self-reported use of blood pressure medication. High serum total cholesterol is defined as serum total cholesterol >7.8 mmol/L. High serum triglycerides is defined as triglycerides >1.7 mmol/L. Low serum HDL cholesterol defined as <1.0 mmol/L (men) and <1.3mmol/L (women)*

**Table S3A:** Mean MoCA score, Z-score (based on available MoCA scores) and VO_2peak_ classified by intervention group among participants with established CVD before inclusion at baseline.

| Year | Control | MICT | HIIT | ExComb | Total |
| --- | --- | --- | --- | --- | --- |
| **Total MoCA score** |  |  |  |  |  |
| Year 3 | 24.0 (3.2) | 25.1 (2.7) | 24.7 (3.3) | 24.9 (3.0) | 24.5 (3.1) |
| Year 5 | 24.1 (3.1) | 24.0 (3.8) | 25.3 (3.1) | 24.5 (3.6) | 24.3 (3.4) |
| Year 10 | 23.7 (3.8) | 25.1 (3.0) | 24.2 (2.3) | 24.8 (2.8) | 24.2 (3.4) |
| **z-score** |  |  |  |  |  |
| Year 3 | -0.84 (1.38) | -0.36 (1.25) | -0.63 (1.38) | -0.48 (1.31) | -0.66 (1.35) |
| Year 5 | -0.81 (1.46) | -0.82 (1.66) | -0.35 (1.41) | -0.63 (1.33) | -0.72 (1.51) |
| Year 10 | -0.71 (1.74) | -0.01 (1.31) | -0.55 (1.09) | -0.01 (1.24) | -0.48 (1.55) |

*Data presented as mean and SD. Total MoCA score is without 1 extra point a recommended by some versions of the test. SD, standard deviation; MICT, moderate-intensity continuous training; HIIT, High-intensity interval training; VO_2peak,_ peak oxygen uptake (mL/kg/min); CVD, Cardiovascular disease.*

**Table S4A:** Linear regression model for normative MoCA scores

|  | **Multiple model**  RC |
| --- | --- |
| **Intercept** | -2.99 |
| **Gender**  Women – ref.  Men | 0  -0.50 |
| **Education**  Compulsory  Secondary – ref.  Tertiary | -0,69  0  1.20 |
| **Age** | 0.83 |
| **Age x Age** | -0.006 |

*Linear regression coefficient used to compute normative MoCA score, modified from Engedal et al.^27.^ RC, Regression coefficient; Ref: Reference.*

# Appendix B

**Adherence to exercise prescription**

Adherence to the prescribed exercise program was assessed using a validated questionnaire^1^ at baseline, and after one, three, five and ten years. The questionnaire covers exercise frequency, intensity, and duration. Frequency was assessed by asking the participants to answer the question “How often do you exercise” with the response options “never” (0 days), “less than once a week” (0.5 days), “once a week” (1 day), “2-3 times per week” (2.5 days) and “almost every day” (5 days). Intensity was assessed by asking the participant to determine their mean intensity of exercise on the Borg scale for ratings of perceived exertion (6-20)^2^. Duration was assessed by asking the participants to answer the question “How long did you exercise each time?” with the response options “less than 15 minutes” (7.5 minutes), “15-29 minutes” (22.5 minutes), “30 minutes to 1 hour” (45 minutes), and “more than 1 hour” (60 minutes).

Adherence in the MICT group was defined as at least 30 minutes of weekly exercise corresponding to 11-14 on the Borg scale. In the HIIT group, the requirements for adherence were at least 30 minutes weekly of exercise at ≥15 on the Borg scale. Requirements for adherence in the control group were ≥75 minutes a week of physical activity.

**Table S1B:** Adherence to the intervention

|  | Control | MICT | HIIT |
| --- | --- | --- | --- |
| **Baseline** |  |  |  |
| - PA activity recommendations | 75% | 78% | 77% |
| - Exercise as MICT | 54% | 53% | 55% |
| - Exercise as HIIT | 22% | 21% | 25% |
| **Year 1** |  |  |  |
| - PA activity recommendations | **80%** | 91% | 91% |
| - Exercise as MICT | 50% | **71%** | 27% |
| - Exercise as HIIT | 25% | 12% | **53%** |
| **Year 3** |  |  |  |
| - PA activity recommendations | **80%** | 88% | 88% |
| - Exercise as MICT | 51% | **67%** | 35% |
| - Exercise as HIIT | 28% | 20% | **51%** |
| **Year 5** |  |  |  |
| - PA activity recommendations | **76%** | 85% | 85% |
| - Exercise as MICT | 50% | **68%** | 39% |
| - Exercise as HIIT | 24% | 15% | **44%** |
| **Year 10** |  |  |  |
| - PA activity recommendations | **72%** | 78% | 77% |
| - Exercise as MICT | 46% | **59%** | 43% |
| - Exercise as HIIT | 23% | 16% | **34%** |

PA, Physical activity; MICT, Moderate intensity continuous training; HIIT, High intensity interval training. Bold numbers indicate adherence to prescribed program.

1. Kurtze N, Rangul V, Hustvedt BE, Flanders WD. Reliability and validity of self-reported physical activity in the Nord-Trondelag Health Study (HUNT 2). Eur J Epidemiol. 2007;22(6):379-87. doi: 10.1007/s10654-007-9110-9.
2. Borg GA. Psychophysical bases of perceived exertion. Med Sci Sports Exerc. 1982;14(5):377-81.
